# Fecal microbiota profiling in irritable bowel syndrome and inflammatory bowel disease patients with irritable bowel syndrome-type symptoms

**DOI:** 10.1186/s12876-021-02015-w

**Published:** 2021-11-19

**Authors:** Xiufang Cui, Haiyang Wang, Ziping Ye, Yi Li, Xinyun Qiu, Hongjie Zhang

**Affiliations:** 1 Department of Gastroenterology, First Affiliated Hospital of Nanjing Medical University, 300# Guangzhou Road, Nanjing, 210029 Jiangsu Province People’s Republic of China; 2 Department of Gastroenterology, Nanjing First Hospital, Nanjing Medical University, Nanjing, Jiangsu Province People’s Republic of China; 3 Department of Gastroenterology, The Affiliated Sir Run Hospital, Nanjing Medical University, Nanjing, 211100 Jiangsu Province People’s Republic of China

**Keywords:** Inflammatory bowel disease, Intestinal microbiota, Irritable bowel syndrome (IBS)-type symptoms

## Abstract

**Background:**

The intestinal microbiota is thought to be involved in the occurrence of inflammatory bowel disease in remission with irritable bowel syndrome (IBS)-type symptoms, but the specific distinct profile of these bacteria remains unclear. This cross-sectional study aims to investigate the fecal microbiota profiling in patients with these diseases.

**Methods:**

Fecal samples from 97 subjects, including Crohn’s disease patients in remission with IBS-type symptoms (CDR-IBS^+^) or without IBS-type symptoms (CDR-IBS^−^), ulcerative colitis patients in remission with IBS-type symptoms (UCR-IBS^+^) or without IBS-type symptoms (UCR-IBS^−^), IBS patients and healthy controls, were collected and applied 16S ribosomal DNA (rDNA) gene sequencing. The V4 hypervariable regions of 16S rDNA gene were amplified and sequenced by the Illumina MiSeq platform. The differences in the sample diversity index in groups were analyzed with R software.

**Results:**

The richness of the intestinal microbiota in the CDR-IBS group was markedly lower than those in the control and IBS groups based on the analysis of observed species and the Chao index (*P* < 0.05). The observed species index in the CDR-IBS^+^ group was higher than that in the CDR-IBS^−^ group (median index: 254.8 vs 203, *P* = 0.036). No difference was found in alpha diversity between UCR patients with IBS-type symptoms and those without related symptoms. At the genus level, the number of *Faecalibacterium* in CDR patients with IBS-type symptoms increased significantly, while *Fusobacterium* decreased versus those without such symptoms (mean relative abundance of *Faecalibacterium*: 20.35% vs 5.18%, *P* < 0.05; *Fusobacterium*: 1.51% vs 5.2%, *P* < 0.05). However, compared with the UCR-IBS^−^ group, the number of *Faecalibacterium* in the UCR-IBS^+^ group decreased, while the number of *Streptococcus* increased, but there was no significant difference in the genus structure. The abundance and composition of the microbiota of IBS patients were not distinct from those of healthy controls.

**Conclusions:**

The IBS-type symptoms in CD patients in remission may be related to an increase in *Faecalibacterium* and a decrease in *Fusobacterium*. The IBS-type symptoms in UC patients in remission cannot be explained by changes in the abundance and structure of the intestinal microbiota.

**Supplementary Information:**

The online version contains supplementary material available at 10.1186/s12876-021-02015-w.

## Background

Changes in the intestinal microbiota can result in the loss of intestinal homeostasis, which has been found in a variety of intestinal disorders, including inflammatory bowel disease (IBD) and irritable bowel syndrome (IBS) [1]. IBD is a chronic relapsing inflammatory disease of the gastrointestinal tract with unknown etiology and includes Crohn’s disease (CD) and ulcerative colitis (UC). The pathogenesis of IBD remains incompletely understood, though it is currently recognized that it is closely related to genetic susceptibility, environment, a disruption of the intestinal microbiota, and immune disorders, especially of the intestinal microbiota. A large number of studies have confirmed that the interaction between intestinal flora and genetic susceptibility can be considered a contributor to the pathogenesis of IBD by triggering an exacerbated immune response [2]. Previous studies indicate that altered gut microbiota composition was found in IBD patients, including reductions in microbial diversity and richness [3]. Specifically, beneficial bacteria decrease, while harmful bacteria increase [4, 5].

Irritable bowel syndrome (IBS) is a functional bowel disease characterized by recurrent abdominal pain, bloating, and altered bowel habits. IBD patients in the active stage often have abdominal pain, diarrhea, bloody stools and other uncomfortable symptoms. Some IBD patients in remission (IBDR) have persistent gastrointestinal symptoms, including abdominal pain, diarrhea and abdominal discomfort. For IBDR, these symptoms meet the criteria for IBS and can be defined as IBS-type symptoms [6–8]. According to previous studies, the prevalence of IBS-type symptoms in patients with IBD with clinically quiescent disease ranges from 25 to 60% due to the different definitions of remission and population sizes [9, 10]. There is a lack of evidence-based therapeutic options available for the management of such patients, who experience a reduced quality of life equivalent to that of patients with overt inflammatory disease activity [10].

Although IBS and IBD are functional and organic diseases, respectively, they have some common etiologies, especially the intestinal microbiome [11, 12]. Emerging evidence suggests an important role of the intestinal microbiota in the pathophysiology of IBS [13, 14]. Previous observational studies have shown that intestinal infections may cause IBS, and probiotics can be used to treat IBS [15, 16]. The etiology of IBS-type symptoms in IBDR patients is still unclear and remains controversial. In addition, at present, there is still a lack of knowledge and effective treatment of the disease in patients with IBDR accompanied by IBS symptoms.

Therefore, we hypothesized that the IBS-type symptoms of IBD patients in remission would be closely related to alterations in the intestinal microbiota. To clarify this hypothesis, we performed a cross-sectional study to initially explore the alterations in the intestinal microbiota of IBD patients in remission with IBS-type symptoms.

## Methods

### Participants and setting

#### IBD patients

All participants with IBD had an established radiological, histological, or endoscopic diagnosis of CD or UC according to the criteria of the European Crohn & Colitis Organization (ECCO) [17]. In this study, IBD patients in clinical remission were included, and the inclusion criteria were as follows [18]: (1) Mayo score ≦ 2 and single-item score < 1; (2) Crohn’s disease activity index (CDAI) ≦ 150; and (3) participants who did not take antibiotics, probiotics, colon cleansing liquids, and similar remedies at least 2 months prior to the study. Exclusion criteria included an inability to understand the Chinese version of the informed consent form, uncategorized IBD, a history of gastroenterology surgery, and female subjects who were lactating or pregnant.

#### IBS and IBD-IBS patients

IBS was evaluated by excluding organic diseases on the basis of Rome IV diagnostic items as follows [19]**.** Patients with confirmed IBD had symptoms of abdominal pain and changes in bowel habits, and these symptoms met the Rome IV criteria [19] and were defined as IBD with IBS-type symptoms (IBD-IBS) according to previous studies [20, 21].

#### Healthy controls

Normal healthy controls were selected from healthy people in the physical examination center. The data were collected from the First Affiliated Hospital of Nanjing Medical University from August 2018 to September 2019.

### Sample collection and genomic DNA extraction

We collected fresh fecal samples from all enrolled subjects and immediately stored them in a -80 °C refrigerator within 2 h to avoid bacterial overgrowth in an oxygen environment. According to the manufacturer’s instructions, genomic DNA for microbiome analysis was extracted using a special QIAamp DNA Stool Mini Kit (Qiagen, Hilden, Germany). Briefly, fecal samples (200 mg) were added to a 2 ml microcentrifuge tube, and tube wea was placed on ice. One milliliter of InhibitEX buffer was added to each fecal sample, and the tube was vortexed continuously for 1 min until the sample was thoroughly homogenized. The subsequent extraction protocol was carried out in strict compliance with recommendations from the QIAamp DNA Stool Mini Kit instructions.

### Sequencing

All DNA samples were quality controlled before being amplified by polymerase chain reaction (PCR) of the V4 hypervariable region of the 16S rRNA gene, a reliable indicator of bacterial taxonomy [22]. Diluted genomic DNA was used as a template, and amplification PCR was performed using specific primers with barcodes, Phusion® High-Fidelity PCR Master Mix with GC Buffer from New England Biolabs, and high-efficiency and high-fidelity enzymes according to the selection of the sequencing region. PCR products were mixed and purified using a Qiagen Gel Extraction Kit. Magnetic beads were used to screen the target amplicon fragments, and finally, the qualified library was used for cluster preparation and paired-end sequencing through the Illumina platform (HiSeq or MiSeq) following the manufacturer’s instructions.

### Data collection and analysis

Samples were merged to build a library using barcodes, and after obtaining clean data, the barcode sequences were used to split the samples through an internally written program. The allowed number of mismatches between barcode sequences and sequencing reads was 0 bp. Using this method, paired-end sequencing was performed on an Illumina platform (HiSeq or MiSeq), and the low-quality reads were removed from the off-machine data. Paired end reads were spliced into tags through the overlap relationship between reads using FLASH (V1.2.11, http://ccb.jhu.edu/software/FLASH/) [23]. The low-quality raw tags were removed, and the high-quality tags remained for subsequent analysis according to QIIME (V1.8.0, http://qiime.org/index.html) [24]. Finally, the obtained effective clean tags were analyzed using the UCHIME algorithm (UCHIME Algorithm, v7.0.1090, http://www.drive5). The software USEARCH (v7.0.1090) was used to cluster the spliced tags into operational taxonomic units (OTUs), with the following procedure: 1) UPARSE was used to perform clustering at 97% similarity to obtain the representative sequences of OTUs; 2) UCHIME (v4.2.40) [4] was used to remove the chimeras generated by PCR amplification from the OTU representative sequence by comparing the sequences with the reference database (Gold database, v20110519, http://drive5.Com/uchime/uchime_download.html; UNITE, v20140703); 3) the Usearch_global method was used to align all tags back to the OTU representative sequences and obtain the abundance statistics table of each sample in each OTU. After obtaining the representative sequences of OTUs, they were compared with the database Greengene_2013_5_99 through RDP Classifier (v2.2) software, the species were annotated, and the confidence threshold was set to 0.6. After the spliced tags were optimized, all samples were selected with the smallest number of tags and clustered into operational taxonomic units (OTUs) for species classification at 97% similarity. In the abundance information, the abundance of OTUs preliminarily indicates the species richness of the sample. In this study, the alpha diversity value of the sample was calculated using mothur (v1.31.2) software. The differences in the sample diversity index in groups were analyzed and displayed with R (v3.1.1) software based on the standardized output data.

### Statistical analysis

All the data were analyzed using Statistical Package for Social Sciences version 25.0 (IBM Company, Armonk, NY). Age parameter data are expressed as the mean ± standard deviation. Unless specifically explained, the majority of microbiota data were nonnormally distributed, and the data are expressed as the median (maximum, minimum). Kruskal–Wallis one-way analysis of variance was used to compare the microbiota data. Partial graphs were drawn using GraphPad software 8.0 (GraphPad Inc., San Diego, CA). A *P* value lower than 0.05 was considered statistically significant.

## Results

### Demographics and clinical characteristics of the study subjects

A total of 97 subjects were enrolled in the study, including 34 IBS patients, 45 IBD patients in remission, and 18 healthy controls. All subjects who met the enrollment criteria from the First Affiliated Hospital of Nanjing Medical University were recruited from August 2018 to September 2019. The mean ages were 42.9 years in the IBS group, 30.9 years in the CDR-IBS + group, 29.8 years in the CDR-IBS- group, 37.1 years in the UCR-IBS + group, 42.4 years in the UCR-IBS- group and 37.9 years in the control group. The proportion of male subjects was 54.6% (53/97). However, there were more female subjects in the IBS group (61.7%, 21/34), which might be closely related to the obvious sex difference in the incidence of this kind of disease. Detailed demographic data and clinical characteristics of all included subjects are listed in Table 1.

**Table 1:** Clinical characteristics of all included patients

	Control	IBS	CDR-IBS +	CDR-IBS-	UCR-IBS +	UCR-IBS-
n	18	34	10	15	10	10
Age, mean ± SD, yr	37.9 ± 8.3	42.9 ± 14.0	30.9 ± 13.9	29.8 ± 10.5	37.1 ± 13.1	42.4 ± 12.7
Sex, male/female	10/5	13/21	5/5	11/4	8/2	8/2
Disease duration, median (range)		12(6–240)	24(12–60)	24(10–120)	27(12–120)	27(9–120)
*Montreal A (Age of onset, yr), n (%)*						
A1 (< 16)			0	1(6.7)		
A2 (17 ~ 40)			8(80)	12(80)		
A3 (> 40)			2 (20)	2(13.3)		
*Montreal A (Location), n (%)*						
L1 (ileal)			3(30)	5(33.3)		
L2 (colonic)			4(40)	3(20)		
L3 (ileocolonic)			3(30)	7(46.7)		
L4 (upper gastrointestinal tract)			0	0		
*Montreal B (Behavior), n (%)*						
B1 (nonstricturing, nonpenetrating)			9(90)	13(86.7)		
B2 (stricturing)			1(10)	1(6.7)		
B3 (penetrating)			0	1(6.7)		
*Montreal E, n (%)*						
E1 (ulceration proctitis)					3(30)	3(30)
E2 (left-sided ulceration colitis)					3(30)	4(40)
E2 (extensive ulceration colitis)					4(40)	3(30)
*Therapy, n (%)*						
5-ASA			2(20)	1(6.7)	8(80)	7(70)
Azathioprine			3(30)	2(13.3)	0(0)	0(0)
Steroids			0(0)	0(0)	2(20)	3(30)
Infliximab			5(50)	11(73.3)	0(0)	0(0)
Nutritional treatment			1(10)	2(13.3)	0(0)	0(0)

CD: Crohn’s disease, CDR: Crohn’s disease in remission, CDR-IBS + : CDR with IBS-type symptoms, CDR-IBS-: CDR without IBS-type symptoms. UC: ulcerative colitis, UCR: ulcerative colitis in remission, UCR-IBS + : UCR with IBS-type symptoms, UCR-IBS-: UCR without IBS-type symptoms; SD: standard deviation; 5-ASA: 5-aminosalicylic acid

### Overall sequencing results

The paired-end reads were optimized to remove low-quality reads and clustered into operational taxonomic units (OTUs) for species classification at 97% similarity, and the abundance information of each sample in each OTU was counted. The abundance preliminarily explains the species richness of the sample. A total of 4,869,075 high-quality tags were obtained, and the average number of tags for each sample was 50,197. According to the 97% similar clustering principle, a total of 1118 OTUs were generated from 97 samples. Compared with the control group, the numbers of OTUs in IBS, CD patients in remission with IBS-type symptoms (CDR-IBS^+^), CD patients in remission without IBS-type symptoms (CDR-IBS^−^), UC patients in remission with IBS-type symptoms (UCR-IBS^+^) and UC patients in remission without IBS-type symptoms (UCR-IBS^−^) were reduced, but only the OTUs of patients in CDR-IBS^−^ had a significant difference (163.7 ± 65.98 vs 240.8 ± 66.75, *P* < 0.05), suggesting that this group of patients may have the lowest species abundance. The detailed results are shown in Table 2.

**Table 2:** The differences of the number of Tags and OUTs

	Control	IBS	CDR-IBS^+^	CDR-IBS^−^	UCR-IBS^+^	UCR-IBS^−^
Tag number	48,231 ± 3293	50,825 ± 2820	48,491 ± 3337	51,135 ± 2373	51,259 ± 4455	50,836 ± 1891
OUT numbers	240.8 ± 66.75	221.4 ± 70.84	225.5 ± 70.48	163.7 ± 65.98*	204.3 ± 79.91	213.2 ± 78.62

CD: Crohn’s disease, CDR: Crohn’s disease in remission, CDR-IBS + : CDR with IBS-type symptoms, CDR-IBS-: CDR without IBS-type symptoms. UC: ulcerative colitis, UCR: ulcerative colitis in remission, UCR-IBS + : UCR with IBS-type symptoms, UCR-IBS-: UCR without IBS-type symptoms. Compared with HC, IBS, CDR-IBS + , ^*^P < 0.05

### Characteristics of the microbial diversity in different groups

**Fig. 1:**
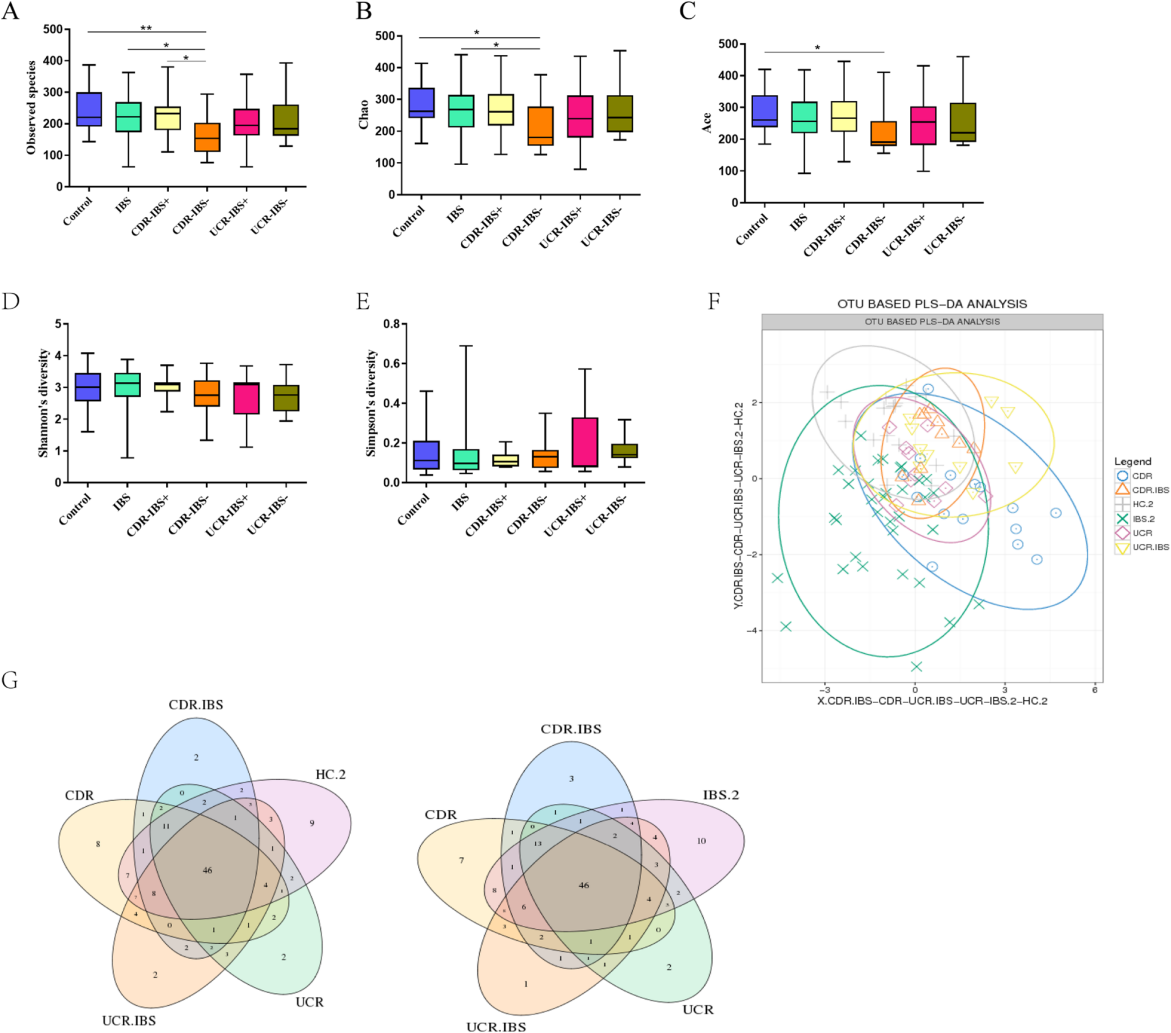
Alpha diversity analysis including community richness (observed species, Chao, Ace) and diversity (Shannon, Simpson) and bacterial community for each group. **A** Observed species; **B** Chao; **C** Ace; **D** Shannon; **E** Simpson; **F** partial least-squares discriminant analysis (PLS-DA) plots; **G** Venn plots. *P < 0.05. *CD* Crohn’s disease, *CDR* Crohn’s disease in remission, *CDR-IBS+* CDR with IBS-type symptoms, *CDR-IBS−* CDR without IBS-type symptoms, *UC* ulcerative colitis, *UCR* ulcerative colitis in remission, *UCR-IBS+* UCR with IBS-type symptoms, *UCR-IBS*− UCR without IBS-type symptoms

A rarefaction curve was used to reflect the rationality of the amount of sequencing data. A rarefaction curve was obtained for each sample according to the number of bacterial OTUs on sequence counts at different sequencing depths. As shown in Additional file 1: Fig. S1, the number of sequences continued to increase, and the rarefaction curve of each sample tended to be saturated, which indicated that the final sequencing data in the study were reliable. Alpha diversity was used to evaluate the differences in the microbiota of samples in the control, IBS, CDR-IBS^+^, CDR-IBS^−^, UCR-IBS^+^, and UCR-IBS^−^ groups. The observed species index, Chao index and Ace index were calculated to reflect the species richness of the microbial community in the sample, while the Shannon index and Simpson index reflected species diversity, which was affected by the species richness and species evenness of the sample community. The results of the comparison among the alpha diversity index groups indicated that the observed species index and the Chao and Ace of CDR-IBS^−^ indexes were significantly decreased compared with those of the control groups, while no difference was found among the other groups (Additional file 1: Fig. S1A–C). Meanwhile, the observed species of microbiota in the CDR-IBS^−^ group was lower than that in the CDR-IBS^+^ group. The Chao index in the CDR-IBS^−^ group was lower than that in the IBS group. Therefore, the richness of microbiota in fecal samples from CDR-IBS^−^ groups was significantly decreased based on the analysis of alpha diversity. There were no differences in the Shannon index and the Simpson index among the six groups (Fig. 1D, E). However, there was no significant difference in the diversity analysis of patients among the IBS, CDR-IBS^+^ and UCR-IBS^+^ groups, including the observed species, Chao1 index and ACE index. Furthermore, the overall microbiota structures were analyzed according to the number of shared or unique OTUs. The results are displayed in partial least-squares discriminant analysis (PLS-DA) plots and Venn plots (Fig. 1F, G) and heat maps at the phylum level. PLS-DA and heat maps indicated that the microbiota structure had slight differences among the groups. However, the majority of the bacterial communities among the six groups overlapped.

### Overall taxonomic compositions of the IBDR, IBS and control groups at the phylum

**Fig. 2:**
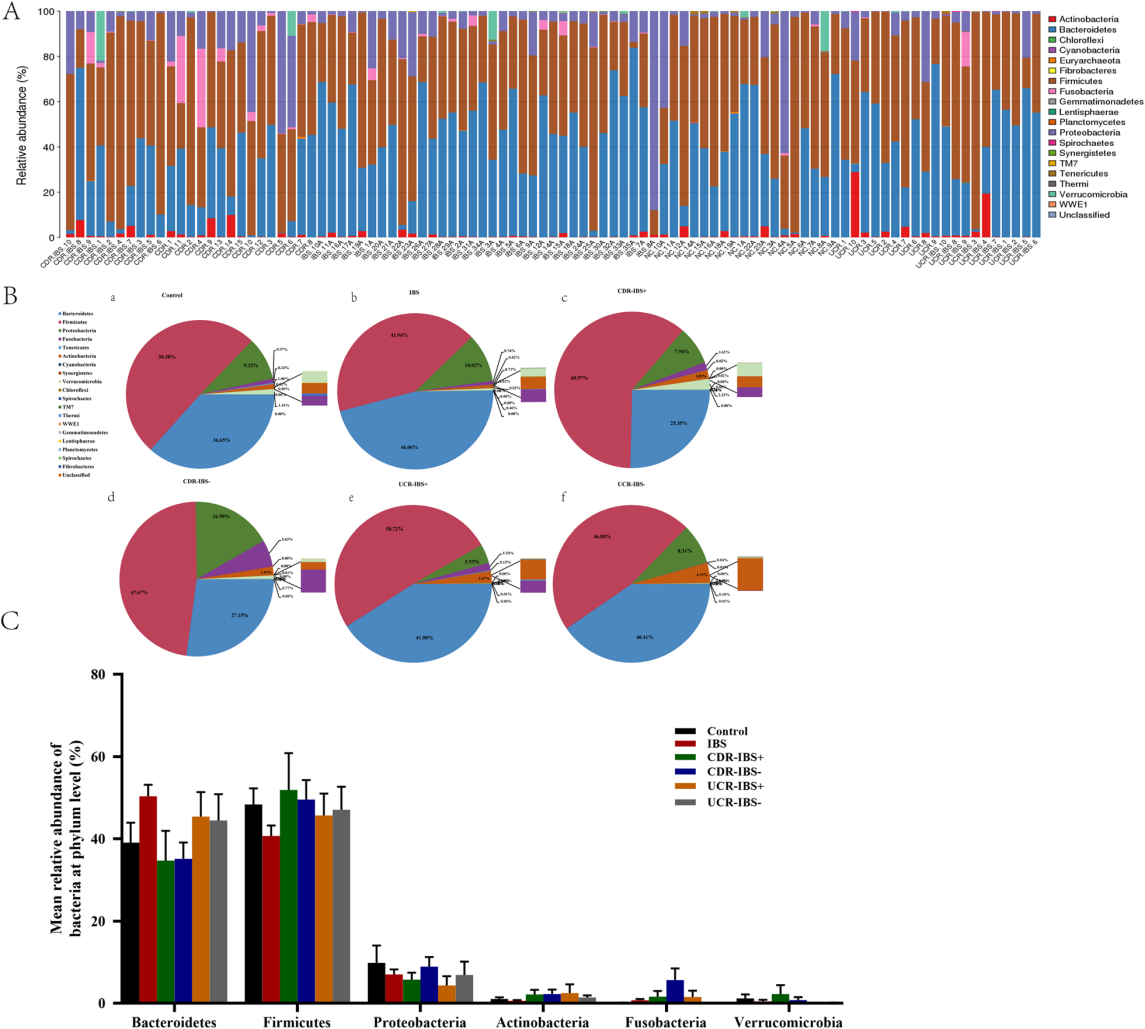
Taxonomic composition of bacteria at the phylum level. **A** Individuals; **B** Integrated chart for the different groups, as follows: a: Control, b: IBS, c: CDR-IBS^+^, d: CDR-IBS^−^, e: UCR-IBS^+^, f: UCR-IBS^−^. **c** The boxplots show the phylum abundances of the 6 most bacterial phyla among the six groups. *CD* Crohn’s disease, *CDR* Crohn’s disease in remission, *CDR-IBS*^+^ CDR with IBS-type symptoms, *CDR-IBS*^−^ CDR without IBS-type symptoms, *UC* ulcerative colitis, *UCR* ulcerative colitis in remission, *UCR-IBS*^+^ UCR with IBS-type symptoms, *UCR-IBS*^−^ UCR without IBS-type symptoms

As shown in Fig. 2, the proportions of different species at the phylum level are summarized in detail for each sample. The phylum levels of taxonomic composition in fecal samples of all groups with major microbiota were as follows: *Firmicutes, Bacteroidetes, Proteobacteria, Actinobacteria, Fusobacteria,* and *Verrucomicrobia.* (Fig. 2A, B). There was no significant difference in microbiota composition at the phylum level among the six groups. Compared with the control group, CDR patients with or without IBS-type symptoms had a decreasing trend in the abundance of *Bacteroidetes*, but there were no statistically significant differences. A relatively decreasing trend in the abundance of *Bacteroidetes* was found in the CDR groups compared with the IBS group (median proportional abundance CDR-IBS^+^ vs IBS: 30.0% vs 47.6%, CDR-IBS^−^ vs IBS: 34.7% vs 47.6%, Fig. 2C), but no significant difference was obtained (*P* > 0.05). The proportion of *Firmicutes* displayed relative increasing trends in the CDR-IBS^+^ and UCR-IBS^+^ groups. No difference in *Fusobacteria* phylum abundance was determined in the current populations, although there was an increasing trend for IBDR in different types regardless of the presence or absence of IBS-type symptoms. Overall, there was no significant difference in microbiota among the IBS, CDR-IBS^+^ and UCR-IBS^+^ groups. Further analysis showed that there was no significant difference in the alteration of the microbiota community at the phylum level between the UCR with or without IBS-type symptoms and the CDR groups.

### Comparison of microbiota composition among the different groups at the genus level

**Fig. 3:**
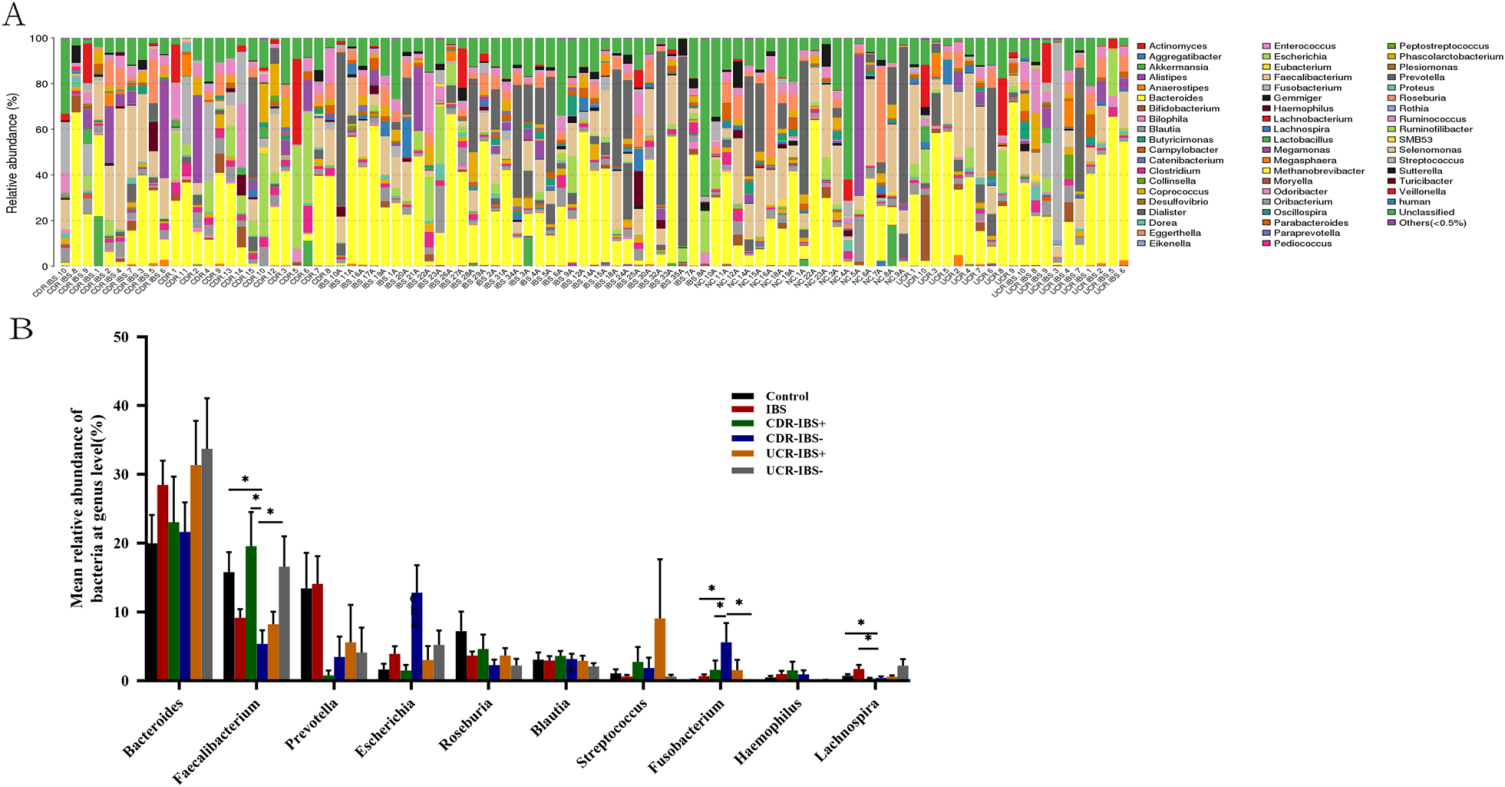
Analysis of taxonomic composition at the genus level. **A** Individually; **B** the boxplot indicates the most abundant bacterial genera in different groups, including the Control, IBS, CDR-IBS^+^, CDR-IBS^−^, UCR-IBS^+^, and UCR-IBS^−^ groups. *P < 0.05. *CD* Crohn’s disease, *CDR* Crohn’s disease in remission, *CDR-IBS*^+^ CDR with IBS-type symptoms, *CDR-IBS*^−^ CDR without IBS-type symptoms, *UC* ulcerative colitis, *UCR* ulcerative colitis in remission, *UCR-IBS*^*+*^ UCR with IBS-type symptoms, *UCR-IBS*^−^ UCR without IBS-type symptoms

The overall genera from each sample are displayed in a bar plot of taxonomic analysis. The main microbiota compositions at the genus level were as follows: *Bacteroides, Faecalibacterium, Prevotella, Escherichia, Roseburia, Blautia, Streptococcus, Fusobacterium, Hemophilus,* and *Lachnospira* (Fig. 3A)*.* There were no significant differences in the changes at the genus level between control and IBS subjects (Fig. 3B). The relative abundances of *Bacteroides* were slightly increased in IBS, UCR-IBS^+^ and UCR-IBS^−^ patients compared to that in controls, but the differences were not statistically significant. The mean abundance of Bacteroides tended to decrease in the CDR-IBS^+^ group and increase in the UCR-IBS^+^ group compared with the IBS group, although the difference was not statistically significant. Compared with CDR-IBS^−^, the abundances of *Faecalibacterium, Roseburia* and *Streptococcus* tended to increase, while that of *Prevotella*, *Escherichia,* and *Fusobacterium* decreased in the CDR-IBS^+^ group, of which the number of *Faecalibacterium* was significantly higher. While that of *Fusobacterium* was lower in CDR-IBS^+^ than in CDR-IBS^−^ (mean relative abundance of *Faecalibacterium*: 20.35% vs 5.18%, *P* < 0.05; *Fusobacterium*: 1.51% vs 5.2%, *P* < 0.05). In addition, the changes in the microbiota community in UCR subjects were not the same as those in CDR subjects. The results indicated that the abundances of *Fusobacterium* and *Streptococcus* were increased*,* but the abundances of *Faecalibacterium, Escherichia,* and *Lachnospira* were slightly decreased in the UCR-IBS^+^ group compared to the UCR-IBS^−^ group, although none of the differences were statistically significant. Differences were also found between CDR and UCR at the genus level. There was a significantly greater abundance of *Faecalibacterium* in UCR-IBS^−^ relative to CDR-IBS^−^(mean proportional abundance 5.4% vs 16.6%, *P* = 0.012) and a greater abundance of *Fusobacterium* in CDR-IBS^−^ relative to UCR-IBS^−^ (mean proportional abundance 5.6% vs 0.04%, *P* = 0.001). The genus differences between CDR-IBS^+^ and UCR-IBS^+^ patients were not statistically significant. The differences in the microbiota communities among the IBS, CDR-IBS^+^ and UCR-IBS^+^ groups were also analyzed. The results showed that the mean abundances of *Faecalibacterium* and *Streptococcus* tended to increase, while the levels of *Prevotella* and *Lachnospira* tended to decrease in the CDR-IBS^+^ and UCR-IBS^+^ groups compared with the IBS groups. However, further analysis of the genera among IBS, CDR-IBS^+^, UCR-IBS^+^ patients did not reveal a statistically significant difference. (Fig. 3B). The differences in genera are displayed in detail in Table 3. As shown, other genera included *Butyricimonas, Odoribacter, Enterococcus, Clostridium, Megasphaera, Ruminofilibacter, Gemmiger, Desulfovibrio, Actinomyces,* and *Akkermansia.* The increasing and decreasing trends of relative abundance in genera are listed in Table 3.

**Table 3:** Significant differences in the microbial distribution of taxa (phylum and genus) in patients with inflammatory bowel disease in remission with IBS-type symptoms

	IBS	CDR-IBS^+^	CDR-IBS^−^	UCR-IBS^+^	UCR-IBS^−^	CDR-IBS^+^ vs UCR-IBS^+^	CDR-IBS^−^ vs UCR-IBS^−^	CDR-IBS^+^ vs IBS	UCR-IBS^+^ vs IBS	CDR-IBS^+^ vs CDR-IBS^−^	UCR-IBS^+^ vs UCR-IBS^−^
***Bacteroidetes***											
*Butyricimonas*	⬆b		⬇a		⬇a				**a**		
*Odoribacter*			⬇b		⬇a						
***Firmicutes***											
*Enterococcus*		⬆b	⬆b	⬆a	⬆a			⬆a			
*Clostridium*		⬆c					**b**				
*Lachnospira*			⬇a				**b**				
*Faecalibacterium*			⬇b				**a**			**b**	
*Megasphaera*		⬆b	⬆b	⬆a	⬆a						
*Ruminofilibacter*				⬆c		**b**			**c**		
*Gemmiger*			⬇**b**								
***Proteobacteria***											
*Sutterella*			⬇a	⬇a							
*Desulfovibrio*			⬇b	⬇b	⬇a				**a**		
***Actinobacteria***											
*Actinomyces*		⬆a	⬆c	⬆a			**b**				
***Fusobacteria***											
*Fusobacterium*	⬆b		⬆c				**b**		**b**		
***Verrucomicrobia***											
*Akkermansia*			⬇a	⬇b		**a**					

⬆ and ⬇ relative to the controls. *CD* Crohn’s disease, *CDR* Crohn’s disease in remission, *CDR-IBS*^*+*^ CDR with IBS-type symptoms, *CDR-IBS*^***−***^ CDR without IBS-type symptoms. *UC* ulcerative colitis, *UCR* ulcerative colitis in remission, *UCR-IBS*^*+*^ UCR with IBS-type symptoms, *UCR-IBS*^***−***^ UCR without IBS-type symptoms. ^a^*P* < 0.05; ^b^*P* < 0.001; ^c^*P* < 0.001

## Discussion

The gut microbiome contains more than 100 trillion different microorganisms, including bacteria, fungi, viruses and protozoa [25]. The majority of the intestinal bacteria belong to four phyla, including *Firmicutes, Bacteroidetes, Proteobacteria* and *Actinobacteria,* and in healthy adults, *Firmicutes* and *Bacteroidetes* are the main phyla [26]. A number of studies have confirmed that changes in the structure and abundance of the intestinal microbiota play important biological roles, including immune regulation, nutrition, metabolism and defense against pathogens [27]. The greater richness and diversity of microbiota are seen as an indicator of good health, while decreased diversity and imbalance in microbiota may be closely related to a large range of diseases, especially intestinal diseases. Studies have confirmed that disorders of intestinal bacteria are involved in the occurrence and development of intestinal diseases, including IBS and IBD [1]. Partial IBD patients in remission may also suffer IBS-like symptoms, which may be related to the intestinal microbiota [28]. However, the results of our cross-sectional study indicate that the onset of IBS might not be related to obvious alterations in intestinal bacteria. Further analysis revealed that CD patients in remission with IBS-type symptoms might be related to an increase in *Faecalibacterium* and a decrease in *Fusobacterium*. There was no statistically significant difference in the abundance of intestinal bacteria between the UCR-IBS^+^ and UCR-IBS^−^ groups at any taxonomic level. UC patients in remission with IBS-type symptoms cannot be explained by changes in the abundance and structure of the intestinal microbiota from our cross-sectional study.

Our study found that the proportion of *Bacteroidetes* in IBS patients at the phylum level tended to increase, while *Firmicutes* decreased compared with control subjects, but the difference was not statistically significant. At the genus level, the alterations in bacterial composition in IBS patients also did not apparently differ from those in controls, while this result was not inconsistent with parts of previous research. Indeed, the role of the fecal microbiota in IBS is still controversial due to different sample sources, study populations, dietary habits and environmental factors. A recent prospective study comparing 110 IBS patients and 39 healthy controls demonstrated that the diversity of fecal microbiota and the number of *Prevotella* were reduced in IBS patients [29]. A systematic review involving microbiota in IBS revealed that the genus *Bacteroides* was increased in IBS patients compared with controls [30]. As shown in Fig. 3B, our results also showed an increasing trend of *Bacteroides* compared with the healthy controls, but the difference failed to achieve statistical significance, which might be explained by the different populations and sample sizes. Consistent with a previous study, our results also found no difference among major phyla or genera between IBS patients and controls [31]. Our results do not support a role for fecal microbiota in the pathogenesis of IBS and correlate with some other studies that reported significant differences between IBS patients and healthy controls in the composition of fecal microbiota [11, 32]. The discrepancy may be explained by different populations, interindividual variation, and no further classification of IBS. Further large-sample cohort studies are needed to confirm the characteristics of the fecal microbiota of IBS patients.

The interaction between the intestinal microbiota and enteric intestinal immune system is the general mechanism of the pathogenesis of IBD, especially in the active stage. Previous studies have confirmed that the disease activity of IBD patients is closely related to a decrease in anti-inflammatory bacterial species and an increase in pro-inflammatory bacterial species, as well as a decrease in overall alpha diversity [33]. However, some patients in IBD remission suffer from varying degrees of IBS-like symptoms [34, 35]. Is there any relationship between the intestinal flora of IBD patients with IBS-like symptoms and that of IBS patients? Therefore, we focused on exploring alterations in the microbiota community and diversity in IBDR patients with IBS-type symptoms. The results indicated that decreased richness (Chao1 and ACE index) was observed in the CDR-IBS^−^ group but not in the CDR-IBS + , UCR-IBS^+^, and UCR-IBS^−^ groups compared with the controls. Furthermore, there were trends toward a decreased number of *Bacteroidetes* and an increased number of *Fusobacteria* in the CDR-IBS^+^ and CDR-IBS^−^ groups based on the analysis of phylum taxonomic levels compared with the control and IBS groups, which might be due to the differences in the diseases. For genus taxonomic analysis, the bacterial community of the fecal sample from CDR-IBS^−^ exhibited an apparent difference from other groups due to the markedly higher number of *Fusobacterium,* an increasing trend of *Escherichia* and lower numbers of *Lachnospira* and *Faecalibacterium* compared to those in CDR-IBS^+^. However, there were no differences in richness and diversity across the CDR-IBS^+^ and CDR-IBS^−^ groups or between the UCR-IBS^+^ and UCR-IBS^−^ groups. A recent study conducted in IBS subjects showed that *Fusobacterium* might exacerbate visceral hypersensitivity [36], which is not consistent with our study. It may be that the study focused on diarrhea-predominant IBS (IBS-D), and we focused on the relationship between IBS symptoms and microbiota during IBD remission. In addition, *Faecalibacterium* belongs to butyrate-producing genera [37] and is elevated in fecal samples of patients with functional bowel disease. Consistent with previous studies, the relative abundance of *Faecalibacterium* was significantly higher than that in the CDR-IBS^+^ group, indicating that the types of genera might play an important role in the formation of IBS-type symptoms. To date, our current results could not provide insight that there is a possible association between the presence of IBS-type symptoms in CD or UC patients in remission. Therefore, it is impossible to comment on any causal relationship between specific microbiome characteristics and the development of IBS-type symptoms, which is consistent with a previous study [21].

Of course, this study had certain limitations. First, this was a cross-sectional study and did not compare the dynamic changes in intestinal bacteria in the development of IBS-type symptoms in IBD patients. In addition, the associated microbiota in mucosa may more accurately reflect the relationship between microbiota and disease, but in our study, only fecal microbiota was detected and analyzed. Second, IBS is clinically divided into several types, including constipation (IBS-C), diarrhea (IBS-D), or a combination of both (IBS-mixed), according to the Rome IV Diagnostic Criteria [38]. The pathogenesis characteristics of the different types of IBS are somewhat different. However, due to the small sample size in this single-center study, no subgroup analysis was performed on the types of confirmed IBS patients or IBS-type symptoms. In addition, this study used the CDAI and Mayo scores to define the active and remission stages of CD and UC, respectively, which are not the gold standards for intestinal inflammation. This is a possible reason for our inability to account for the significant alteration of microbiota in CDR-IBS^−^.

Our study and a previous study [21] failed to detect any difference in CDR-IBS composition and diversity in IBDR patients reporting IBS-type symptoms. However, clinical trials including probiotics and low fermentable oligosaccharide, disaccharide, monosaccharide, and polyol (FODMAP) diets have obtained promising results in IBS, indicating the role of the intestinal microbiota [39, 40]. Additionally, the effects of probiotics and fecal bacteria transplantation (FMT) in the treatment of IBD have multiple benefits, and no attention is focused on the impact of these treatments on IBD-like symptoms [41, 42].

## Conclusions

In conclusion, the obtained results from our study did not find any difference in the intestinal microbiota between IBD patients in remission with IBS-type symptoms and those without IBS-type symptoms. These results provide a certain basis for recommendations for future trials on the management of IBS-type symptoms. In the future, we still need to have a better understanding of the mechanism of IBD with IBS-type symptoms in the absence of persistent disease activity and strive to find effective treatments to relieve clinical symptoms and improve life treatment.

## Supplementary Information

**Additional file 1: Figure S1**. Rarefaction analysis of sampling by observed bacterial.

## Data Availability

The original data supporting this research conclusion are available from the corresponding author.
