# Supplementary material for: Fecal microbiota profiling in irritable bowel syndrome and inflammatory bowel disease patients with irritable bowel syndrome-type symptoms
Source: BMC Gastroenterol. 2021 Nov 19;21:433. doi: 10.1186/s12876-021-02015-w (PMC8603515; doi:10.1186/s12876-021-02015-w)

**Fecal microbiota Profiling in Irritable Bowel Syndrome and Inflammatory Bowel Disease Patients with Irritable Bowel Syndrome-Type Symptoms**

Xiufang Cui^1#^, Haiyang Wang^2#^, Ziping Ye^1^, Yi Li^2^, Xinyun, Qiu^1^, Hongjie Zhang^1^*

1 Department of Gastroenterology, First Affiliated Hospital of Nanjing Medical University,

Nanjing, Jiangsu Province, China

2 Department of Gastroenterology, The Affiliated Sir Run Run Hospital, Nanjing Medical University, Nanjing 211100, Jiangsu Province, China

***Correspondence to:** Dr. Hongjie Zhang, Department of Gastroenterology, First Affiliated Hospital of Nanjing Medical University, 300# Guangzhou Road, Nanjing 210029, Jiangsu Province, P.R. China; Phone: 0086-(0) 25-8371-8836, Ext. 6973 or 6920; Fax: 0086-(0) 25-83674636; E-mail: [hjzhang06@163.com](mailto:hjzhang06@163.com).

# These authors contributed equally to this article.

Supplementary Figure S1: Rarefaction analysis of sampling by observed bacterial


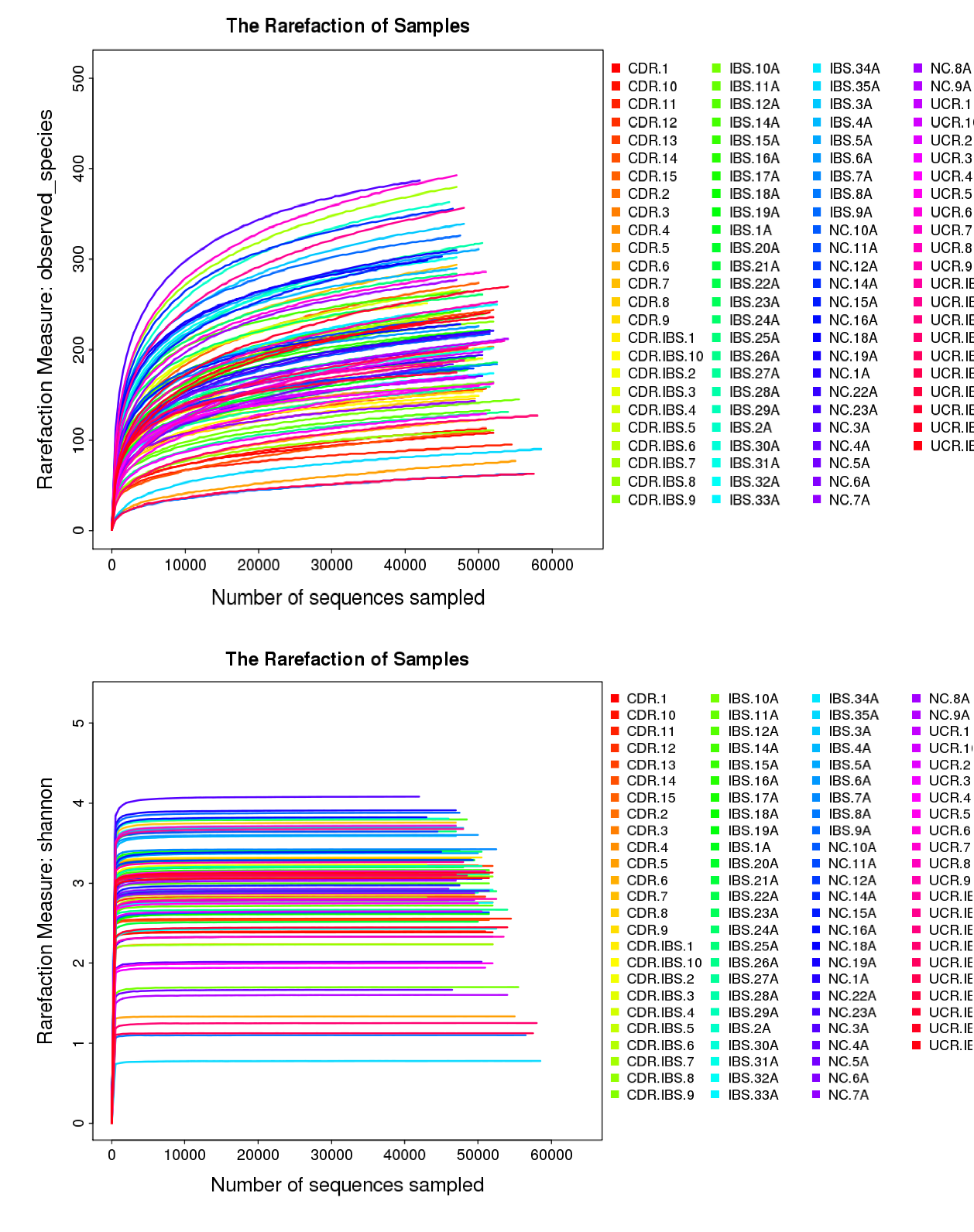

Supplement: Supplementary file 1 — Additional file 1: Figure S1. Rarefaction analysis of sampling by observed bacterial. [file 12876_2021_2015_MOESM1_ESM.docx]
